# Social environment shapes female settlement decisions in a solitary carnivore

**DOI:** 10.1093/beheco/arab118

**Published:** 2021-10-18

**Authors:** J E Hansen, A G Hertel, S C Frank, J Kindberg, A Zedrosser

**Affiliations:** 1 Faculty of Technology, Natural Sciences and Maritime Sciences, Department of Natural Sciences and Environmental Health, University of South-Eastern Norway, Bø i Telemark, Norway; 2 Senkenberg Biodiversity and Climate Research Centre, Frankfurt, Germany; 3 Norwegian Institute for Nature Research, Trondheim, Norway; 4 Swedish University of Agricultural Sciences, Department of Wildlife, Fish, and Environmental Studies, Umeå, Sweden; 5 Department of Integrative Biology, Institute of Wildlife Biology and Game Management, University of Natural Resources and Applied Life Sciences, Vienna, Austria

**Keywords:** dispersal, public information, settlement, social environment, space use

## Abstract

How and where a female selects an area to settle and breed is of central importance in dispersal and population ecology as it governs range expansion and gene flow. Social structure and organization have been shown to influence settlement decisions, but its importance in the settlement of large, solitary mammals is largely unknown. We investigate how the identity of overlapping conspecifics on the landscape, acquired during the maternal care period, influences the selection of settlement home ranges in a non-territorial, solitary mammal using location data of 56 female brown bears (*Ursus arctos*). We used a resource selection function to determine whether females’ settlement behavior was influenced by the presence of their mother, related females, familiar females, and female population density. Hunting may remove mothers and result in socio-spatial changes before settlement. We compared overlap between settling females and their mother’s concurrent or most recent home ranges to examine the settling female’s response to the absence or presence of her mother on the landscape. We found that females selected settlement home ranges that overlapped their mother’s home range, familiar females, that is, those they had previously overlapped with, and areas with higher density than their natal ranges. However, they did not select areas overlapping related females. We also found that when mothers were removed from the landscape, female offspring selected settlement home ranges with greater overlap of their mother’s range, compared with mothers who were alive. Our results suggest that females are acquiring and using information about their social environment when making settlement decisions.

## Teaser

Information about the social environment may help female brown bears to select a settlement home range for breeding. We found that a female uses the identity of other females that overlapped her natal home range and female density when making settlement decisions. Specifically, females select settlement home ranges that overlap with home ranges of their mothers and familiar females known from their natal period. Relatedness does not appear to influence settlement decisions in this population.

## INTRODUCTION

How and where a female selects an area to settle and breed is of central importance in dispersal and population ecology (Pulliam and Danielson 1991; Barton 1992; Stamps 2001). After a variable amount of time living in a natal home range (NHR), selection of a settlement area is the final stage in natal dispersal, that is, dispersal before breeding (Bowler and Benton 2005). A common settlement pattern among mammals is for subadult males to disperse and for females to remain philopatric, that is, settle where they overlap their NHR (Waser and Jones 1983). However, even in mammals with general female philopatry it is common for some females to disperse (Lawson Handley and Perrin 2007), and such plasticity in dispersal pattern suggests behavioral control over the settlement process (Benard and McCauley 2008).

Proximate cues in the natal period may influence where individual females settle (Stamps 2001; Benard and McCauley 2008). Natal habitat preference induction is a mechanism whereby individuals use environmental cues from their NHR when searching for settlement areas (Stamps and Davis 2006). Another mechanism is density dependence (Matthysen 2005), that is, females living at higher population densities may be limited in areas available to settle and breed (Fretwell and Lucas 1969; Stockley and Bro-Jørgensen 2011). Kin-based dispersal, that is, females settle in areas away from related individuals to avoid kin competition and thereby increase their inclusive fitness, has also been proposed as a mechanism for female settlement decisions (Cote and Clobert 2010). Conversely, in other social systems females settle where they overlap kin with increased tolerance towards related individuals, which may also increase inclusive fitness (Clutton-Brock and Lukas 2012).

The influence of social information, that is, information acquired by observing other individuals interact with the environment (Danchin 2004), on the settlement process is receiving attention as a mechanism influencing settlement patterns (Vercken et al. 2012; Wey et al. 2015). The use of social information for settlement decisions has been described for a variety of taxa (Doligez et al. 2002; Nocera et al. 2006; Robinson et al. 2011; Vercken et al. 2012), but has received less attention in mammals (Valone 2007). This implies that social structure (relationships and interactions among individuals) and social organization (the composition of individuals within an area or group), that is, the social environment, can provide information for individual dispersal decisions (Bowler and Benton 2005; Armansin et al. 2020). For example, conspecific density can be used to assess the quality of a resource or an environment (Dall et al. 2005). In addition, information about the social environment gleaned in the natal period, for example, the presence and location of conspecifics on the landscape (Danchin et al. 2004) or the detection of a female with dependent young (Clobert et al. 2001), can provide naïve individuals with valuable information, for example, on habitat availability or reproductive competition, and reduce uncertainty for their settlement decisions (Danchin et al. 2004). Lastly, the use of information regarding specific female identities allows an individual to gain familiarity with overlapping conspecifics which can reduce ‘social resistance’ into an area for settlement (Armansin et al. 2020).

Research on the influence of the social environment in mammals is typically conducted on social species (Hare et al. 2014; O’Mara et al. 2014). We use the brown bear (*Ursus arctos*) as model species to investigate if the social environment influences natal dispersal and the selection of settlement home ranges (SHR) in a non-territorial, solitary mammal. Brown bears live solitarily for the majority of their lives aside from the mating period and females rearing offspring. Adult females maintain relatively stable home ranges with extensive spatio-temporal overlap, especially among related individuals (Mace and Waller 1997; Støen et al. 2005). Female brown bears likely exhibit inverse density dependent dispersal, which may result in the formation of matrilineal assemblages, that is, overlapping home ranges of several generations of related females (Støen et al. 2005). Reproductive suppression has been documented in female brown bears, and it has been suggested that related females (Støen et al. 2006b) and neighboring females (Ordiz et al. 2008) influence one another’s breeding patterns. After the death of an adult female, other females will shift their home ranges to fill in that vacancy (Frank et al. 2017). This suggests that female brown bears may make decisions on space use and reproduction based on information regarding the social environment. Information gathering from conspecifics may reduce uncertainty in the settlement process and result in an increased chance of successful breeding (Danchin et al. 2001). Brown bears extensively use chemical scent cues (Clapham et al. 2012, 2014; Jojola et al. 2012; Morehouse et al. 2021) and other spoor on the landscape (Sergiel et al. 2017), and scent communication is the most likely means of acquiring information on conspecifics (Revilla et al. 2021). Additionally, information about the social environment may be obtained through direct social interactions with spatially overlapping females. We propose that females acquire information about the social environment during the natal period, such as the identity and density of conspecifics, that is later used in the selection of an SHR.

The primary objective of this study is to investigate if the social environment influences selection of an SHR by a solitary-living mammal, the brown bear. We hypothesized that, amongst all female conspecifics, an individual female’s mother would exhibit the highest level of social tolerance towards them. We thus predicted that (P1) females would select SHR that overlapped their mother’s home range. However, hunting in the population leads to regular socio-spatial changes (Frank et al. 2017), and a mother may die before a female’s settlement decision. We hypothesized that females detect the presence or absence of their mother on the landscape and would use that information when making settlement decisions. Specifically, we predicted that (P2) females whose mothers died before settlement would overlap a greater amount of their mother’s home range than females whose mothers were alive in the settlement period. A kin-based socio-spatial structure has been documented in female brown bears (Støen et al. 2005) and we hypothesized that females recognize and show higher social tolerance to kin than non-kin. We thus predicted that (P3) females would select SHR that overlapped related females. The presence of females who are familiar from the natal period (Mateo 2002) may decrease aggressive encounters between neighboring females and improve breeding success (Ylönen et al. 1990; Armansin et al. 2020). We hypothesized that during the natal period females gained familiarity with neighboring females who may be less hostile to a female in the settlement period. We, therefore, predicted that (P4) female bears would select SHR that overlapped with females “known” from the natal period, hereafter “familiar” females. Previous research on female brown bears indicated that density patterns are regulated through social interactions of females and that individuals have the ability to detect local density levels and that reproductive success of primiparous females is higher in lower density areas (Støen et al. 2006a; Zedrosser et al. 2009). We hypothesized that females detect density levels of conspecifics in the natal period and during the selection of an SHR. We, therefore, predicted (P5) females would select an SHR with a density lower relative to the NHR to reduce competition.

## MATERIALS AND METHODS

### Study area and model species

Our study area covers ~13,000 km^2^ and is located in Gävleborg and Dalarna counties, southcentral Sweden (~61ºN, 14ºE). The terrain is hilly, and Scots pine (*Pinus sylvestris*) and Norway spruce (*Picea abies*) are the dominating tree species. The forests are intensively managed, resulting in a mosaic of mixed-aged stands, bogs, and lakes. The human population density in the region is low (Ordiz et al. 2012), but there exists an extensive network of forestry roads (Frank et al. 2015). Bears are hunted in the study area (Frank et al. 2017).

Brown bears are solitary-living and non-territorial carnivores with a promiscuous mating system and extensive overlap of male and female home ranges (Støen et al. 2005; Steyaert et al. 2012). The mating season is from May to July (Steyaert et al. 2012) and females give birth to cubs during hibernation in January (Friebe et al. 2001). Offspring in the Scandinavian population remain with their mothers in the NHR for either 1.5 or 2.5 years until family break up (Van de Walle et al. 2018). Dispersal is a gradual process over a 2–3 year timespan, and up to 54% of the female subadults will settle in areas overlapping their NHR (Støen et al. 2006a). The average age of female primiparity in this population is 5 years (Zedrosser et al. 2009).

### Telemetry data and home range estimation

The population in the study area has been continuously monitored since 1985 (Swenson et al. 1994). Very-high frequency (VHF) telemetry data span from 1985 to 2016 and Global Positioning System (GPS) telemetry data from 2003 to 2018 (Dahle and Swenson 2003; Arnemo and Evans 2017). Capture and handling of bears was conducted by permit under Swedish authorities and ethical committees (Uppsala Djurförsöksetiska Nämnd: C40/3, C212/9, C47/9, C210/10, C7/12, C268/12, C18/15. Statens Veterinärmediciniska Anstalt, Jordbruksverket, Naturvårdsverket: Dnr 35-846/03, Dnr 412-7093-08 NV, Dnr 412-7327-09 Nv, Dnr 31-11102/12, NV-01758-14). Relocations from VHF collars were obtained weekly on average and GPS collar data were collected hourly. We inspected VHF locations for outliers and removed locations that occurred beyond the average home range diameter (14 km) from the cluster of locations of a given individual. To minimize location error of GPS relocation data, we removed GPS locations with a dilution of precision (DOP) > 10 from the data (D’eon and Delparte 2005). We then subset the screened telemetry data to the average local active period for bears, 01 May to 30 September (Friebe et al. 2001).

We generated annual 95% kernel utilization distributions (UD) using the R package *adehabitatHR* (Calenge 2006) for each focal bear. A focal bear’s NHR was estimated from the UD of its mother in the year before family breakup, as focal bears had not yet received radio collars during this time period. We estimated a focal bear’s SHR from its own annual UD two years after family breakup, before primiparity (Zedrosser et al. 2009). We selected two years post family breakup because previous work has shown peak dispersal activity for females in the year after family break up (Støen et al. 2006a) and our goal was to best estimate where females settled. For some focal bears (*n* = 24), telemetry data were unavailable for the second year after family breakup. In these cases, we used the first available year post breakup (from 1 to 4 years) for the SHR. For females with living mothers during the settlement period, we estimated the mother’s home range in the focal bear’s settlement year. For females whose mothers died before settlement, we estimated the mother’s home range in the most recently available year (1–2 years before settlement). We extracted the 95% vertices of each annual UD to generate home ranges. We calculated the centroid of the NHR and SHR of each focal individual.

Between 50 and 80% of female bears in the study area are marked on an annual basis (Bellemain et al. 2005; Zedrosser et al. 2006), but not all marked bears have adequate relocation data for home range estimation. To approximate the home ranges of non-focal adult (≥4 years of age) females (*n* = 1259 bear years), we used varying methods based on the availability of their location data. We used the available location as an approximated “home range” centroid for each year of the adult lifespan (≥4 years of age) for marked females with only a single geolocation, for example, the location of their capture or mortality. For marked females with too few locations to estimate a UD, we obtained the centroid of all available points to serve as the annual “home range” centroid. For females with adequate location data, we estimated the 95% annual UD and obtained a home range centroid. We completed a sensitivity analysis to determine the appropriate buffer size around all non-focal female centroids to create assumed home ranges. We overlapped circular polygons with radii from 6–15 km with female home ranges from our study population with the goal of maximizing home range coverage and minimizing the amount of buffer extending beyond the home range. A buffer size of 10.5 km was large enough for 95% average coverage of all known female home ranges although exceeding home ranges by only 5% on average.

We obtained telemetry data from 56 dispersing females and 31 unique mothers between 1998 and 2018; each represented in the natal and settlement year by ≥20 VHF (range = 20–151, mean = 80) or ≥1000 GPS (range = 1045–6913, mean = 3543) locations. Local density varied from one to 15 females (mean = 7 ± 4 SD) in the NHR and from zero to 11 females (mean = 5 ± 3) in the SHR (see Table S1 for further variable information).

### Defining the social landscape

**Figure 1:**
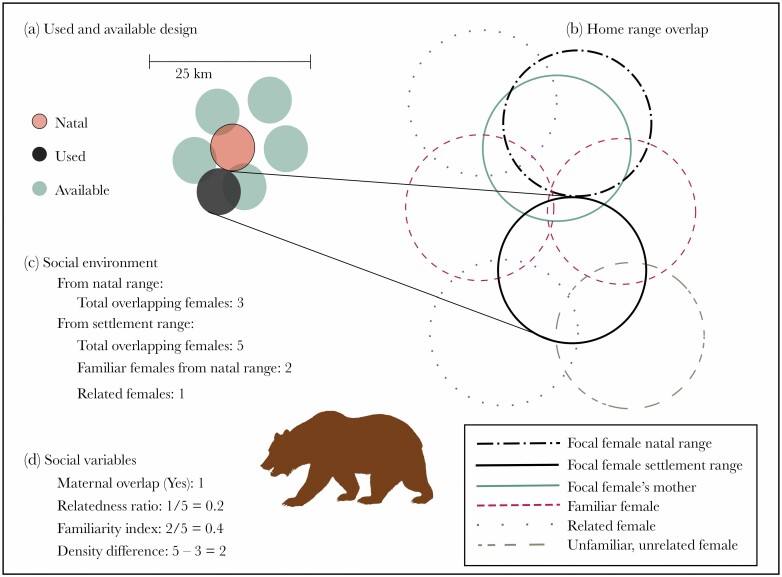
Schematic of how social variables were derived for the study on the influence of social environment on female brown bear settlement home range (SHR) selection. a) Used and available SHR design for the resource selection function. For each used SHR, 5 random available home ranges are created within 25 km of the natal home range. b) A focal female’s natal home range (NHR) and each used and available settlement home range were overlaid with the home ranges of all other females on the landscape. c) Social environment variables were extracted based on the overlapping females, including the density (total overlapping females) in the NHR and SHR, the number of related and known females overlapping the SHR, and whether the focal female’s SHR overlapped her mother’s home range. d) Four social variables (maternal overlap, relatedness ratio, familiarity index, and density difference) were derived based on social environment values extracted in b. Example calculations are given below each (c) and (d).

We identified female focal individuals that had available telemetry data in their natal and settlement years. We overlapped the NHR and SHR of focal individuals with the home ranges of non-focal females in each natal and settlement year (Figure 1). To test for predictions 1 and 3–5, we generated four variables based on the resulting overlap data to represent the social landscape (Figure 1). *Maternal overlap* is a binary yes/no variable of whether or not the home range of the focal bear’s mother overlapped their own SHR. This variable is only relevant for focal bears who have living mothers in the settlement year. *Relatedness ratio* in the SHR represents the proportion of related females among all overlapping females. We took tissue and hair samples from captured or dead bears in the population and used 16 microsatellites to genotype 1614 individuals. We constructed pedigrees and used genotypes of individuals to assign relatedness of females (detailed in Frank et al. 2020) with the Lynch–Ritland estimator (Lynch and Ritland 1999). This method allows for the estimation of the coefficient of relatedness using molecular markers. We used a pairwise coefficient of relatedness value of ≥0.125 as the threshold for two females to be related. This threshold includes up to third order relationships such as a great grandmother, aunt, or cousin. If all females overlapping the SHR were unrelated, we assigned zero as the relatedness ratio. We did not include a focal bear’s mother in relatedness ratio due to her being represented already in the maternal overlap variable. *Familiarity index* represents females that overlapped the NHR (hereafter “familiar”). We divided the number of familiar females by total females overlapping the focal individual’s SHR to generate the familiarity index. If no familiar females overlapped the SHR, we assigned zero. We did not include a focal bear’s mother in the familiarity index due to her existing representation in the maternal overlap variable. *Density difference* is the difference in local female density (total number of overlapping females) between the SHR and NHR. Obtaining the difference in density allowed us to incorporate variation in natal density among the focal females, as it represents the change in density from the NHR towards the SHR.

To test prediction 2, that is, the effect of the mother being alive or dead during the settlement period, we overlapped the SHR of the focal female with her mother’s HR in the settlement year or most recent available year. We calculated an index of overlap for a focal female’s SHR and her mother’s HR using the following formula:


$$\begin{array}{l} \left(O_{ij}/\left(A_{i}+A_{j}\right)\right)*2, \end{array}$$

where O_ij_ represents the area of overlap between the mother and focal female’s home ranges, A_i_ is the total area of the mother’s home range, and A_j_ is the total area of the focal female’s home range (Støen et al. 2005); overlap index values are between 0 (no overlap) and 1 (complete overlap).

### Statistical analyses and modeling approach

To address our primary objective, the influence of the social environment in settlement decisions, we used second order resource selection functions (RSF) with a used-available design and an exponential selection function (Manly et al. 2002), which represents selection at the level of individual home ranges (Johnson 1980). The ‘used’ locations were the set of SHR centroids for the focal females. For each used location, we generated 5 “available” locations representing potential home range centroids where an individual could have settled (Figure 1). We based availability on empirical dispersal distances from the NHR for our focal females (range 1.3–67.7 km). We selected 25 km as the upper limit on availability as this represents the 95% distribution of distances between the NHR and SHR and reflects the average dispersal distance for females in this population (Støen et al. 2006a). We attempted to control for distance by fitting a probability distribution to the dispersal distances and randomly selecting 5 available locations weighted by probability within that buffer using the *spsample* function in the *sp* package (Pebesma and Bivand 2005). We buffered each used and available centroid by 10.5 km to generate a series of used and available SHR polygons from which we extracted our social variables (Figure 1). We used the *glmmTMB* package (Brooks et al. 2017) to fit a logistic generalized linear mixed model with the binary response variable of used (1) and available (0) SHR. As predictor variables, we used *maternal overlap*, *relatedness ratio*, *familiarity index*, *density difference,* and a random intercept for focal individual (focalID). We included an additional random intercept for the number of years since family break up (1–4) to account for variability among individuals. We standardized all continuous predictor variables to have a mean of zero and a standard deviation of one. Due to the importance of habitat features for survival (DeCesare et al. 2014; Matthiopoulos et al. 2015), we initially included the following habitat covariates (measured as percent cover over the home range) in our model: built (anthropogenic) environment, cultivated land, mature forest, young forest, clearcuts, and bogs (see Supplementary S1 for details).

Before modeling, we checked all predictor variables for collinearity by verifying that all variables had variance inflation factors (VIF) < 2. We conducted further analyses investigating the relationship between relatedness ratio and familiarity index because there was potential overlap in those two variables (Supplementary S2). We first fit a base model that included all social predictor variables and no interactions and then sequentially fit the base model containing one interaction term for each combination of predictor variables. We fit only one interaction term per model due to sample size limitations. We did not have a priori hypotheses regarding the relative importance among the social variables, so we fit the base model and fit all possible subsets of the base model using the *MuMIn* package (Bartoń 2018) and ranked them according to Akaike’s Information Criterion adjusted for small sample sizes (AICc, Hurvich and Tsai 1995). We retained models within ΔAICc ≤ 2 of the highest-ranked model for further assessment and interpretation (Burnham and Anderson 2004). We calculated Nakagawa’s pseudo *R*^2^ (Nakagawa and Schielzeth 2013) to evaluate goodness of fit and explanatory power. We averaged coefficients and 95% confidence intervals (CI) over the retained model set for each variable. CIs containing zero were considered uninformative (Arnold 2010).

We employed a bootstrapping modeling procedure to assess relative variable importance. For each permutation, we randomly sampled with replacement observations from 90% of the focal bears in the study. In each permutation, we fit the base model and all possible subsets, ranked them via AICc, and retained those with ΔAICc ≤ 2 (Grueber et al. 2011). We then averaged each set of top models and summed model weights by variable, that is, whether a focal variable was contained in the model. After 1000 permutations, we took the mean value of each variable’s summed Akaike weight to measure the relative importance among the social variables (Galipaud et al. 2014).

To assess how the mother’s presence on the landscape influences settlement decisions, we compared the overlap index between females whose mothers were alive during settlement with those whose mothers died before that period with a Wilcoxon test. One female in each group had overlap values of zero, so we removed them from the analysis to avoid “ties.” We performed all statistical analyses in R 3.6.2 (R Core Team 2019).

## RESULTS

We did not detect selection or avoidance for any habitat features at the second order scale and model selection showed the highest support for the model containing only social variables (see Supplement S1 for a detailed description on methodology and results from habitat modeling). We did not detect any informative interactions between the predictor variables (Table S2), and the base model received greater support than any models containing an interaction term. We also did not see an effect in the random intercept for a year since the family breakup (Table S3) and the base model received higher support than the model with the additional random intercept.

**Figure 2:**
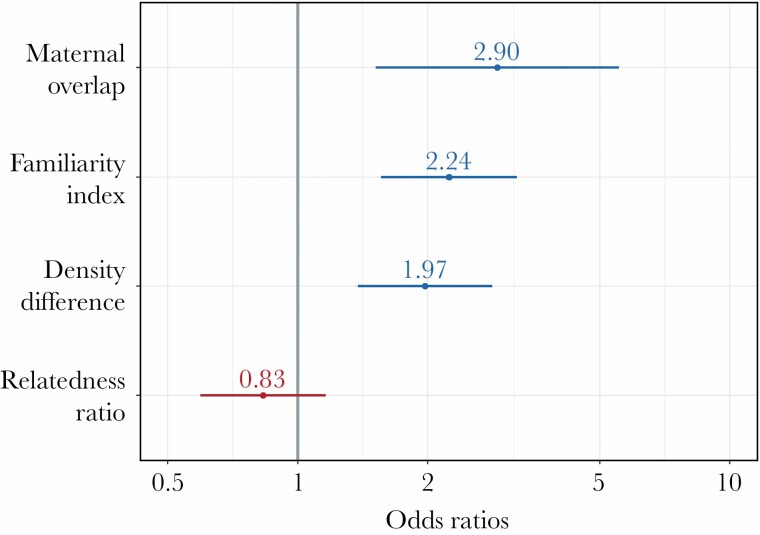
Plot of fixed effect coefficient estimates with 95% confidence intervals (coefficients were exponentiated to derive odds ratios) from a resource selection function averaged model estimating the influence of social variables on female brown bear selection of settlement home ranges in Scandinavia (from 1998–2018). Fixed effects were standardized to a mean of zero and a standard deviation of one. Variables with confidence intervals not overlapping one are considered informative. Exponentiated 95% confidence interval values are 1) maternal overlap (1.51–5.54), 2) familiarity index (1.56–3.22), 3) density difference (1.37–2.82), and 4) relatedness ratio (0.59–1.16).

Two models had ΔAICc ≤ 2; each of the predictor variables appeared in at least one top model (Table 1). Akaike weights of the top models indicated no clear support for a single model, so we present coefficients from the averaged model (Figure 2). Female bears selected for SHR that overlapped mothers (β = 1.03, 95% CI: 0.3–1.67), familiar females (β = 0.8, CI:0.44–1.17), and areas with higher density relative to their natal range (β = 0.67, CI: 0.32–1.03), but were indifferent for overlap with related females (β = −0.19, CI: −0.52to 0.15). The results of the model bootstrapping procedure indicated that *maternal overlap, density difference,* and *familiarity index* had the highest variable importance (Table 2). In contrast, the CI of *relatedness* ratio contained zero and had the least relative importance in the bootstrapping procedure (Table 2).

**Table 1:** Resource Selection Function model results showing the influence of the social landscape on female brown bear selection of settlement home ranges in Sweden, 1998–2018. Model selection table shows the two most supported models (highest-ranked model and others ∆AICc < 2) plus the null model. Values shown are the degrees of freedom, log likelihood, AICc, ∆AICc, model weight, and Nakagawa’s Pseudo *R*^2^ for the marginal (fixed effects) model. Social variable codes are as follows: densDiff = difference in density from SHR to NHR, famIx = familiarity index, matOver = maternal overlap, relRatio = relatedness ratio

Model set	K	logLik	AIC_c_	ΔAIC_c_	W_i_	Pseudo *R*^2^
densDiff + famIx + matOver	5	−131.72	273.62	0.00	0.6	0.27
densDiff + famIx + matOver + relRatio	6	−131.11	274.48	0.86	0.39	0.27
Null model	2	151.39	306.81	33.2	0	0

**Table 2:** Results of averaging the top models of the influence of the social landscape on female brown bear settlement home range selection in Scandinavia (between 1998 and 2018). Summary of parameter estimates, standard error, and 95% confidence interval (CI) after model averaging each covariate on probability of use for used and available settlement home ranges. Relative importance of each variable is from their summed Akaike weights. Variable codes are as follows: matOver = maternal overlap, famIx = familiarity index, densDiff = change in density from settlement to natal home range, relRatio = relatedness ratio

Variable	β	SE	CI	Relative importance
matOver	1.03	0.32	0.33–1.67	0.95
famIx	0.8	0.19	0.44–1.17	0.99
densDiff	0.67	0.18	0.32–1.03	0.99
relRatio	−0.19	0.17	−0.52 to 0.15	0.34

**Figure 3:**
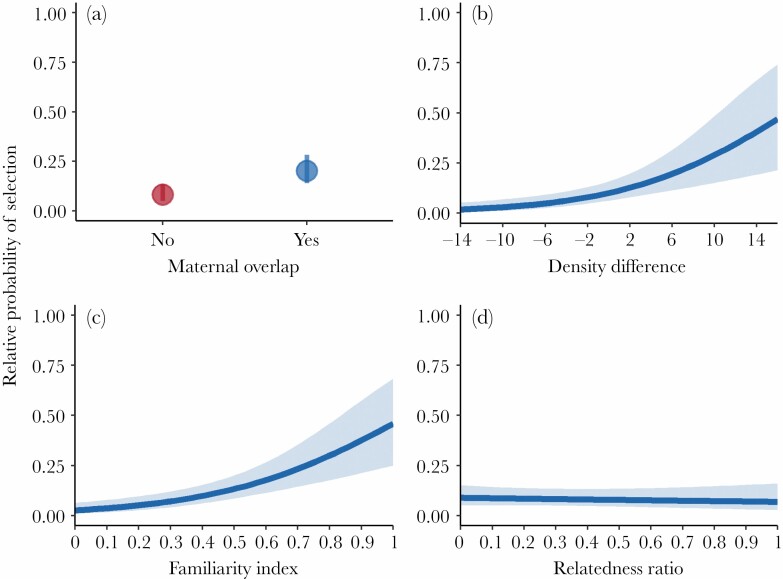
Relative probability of a female brown bear selecting a settlement home range (SHR) dependent on a) overlapping her mother’s home range, b) the difference in female bear density in the SHR relative to her natal home range, c) the proportion of familiar (individuals a focal female had natal home range overlap with) to total females overlapping the SHR, and d) the proportion of related females to total females overlapping the SHR. Data are predicted from a resource selection model for the female Scandinavian brown bear population between 1998–2018.

Probability of selection for SHR increased with maternal overlap, presence of familiar females, and areas with higher density relative to their natal range, but not for related females (Figure 3). Pseudo R^2^ values for the top models indicated that modeling social factors alone explains 27% of variation in second order settlement patterns for female bears (Table 1).

**Figure 4:**
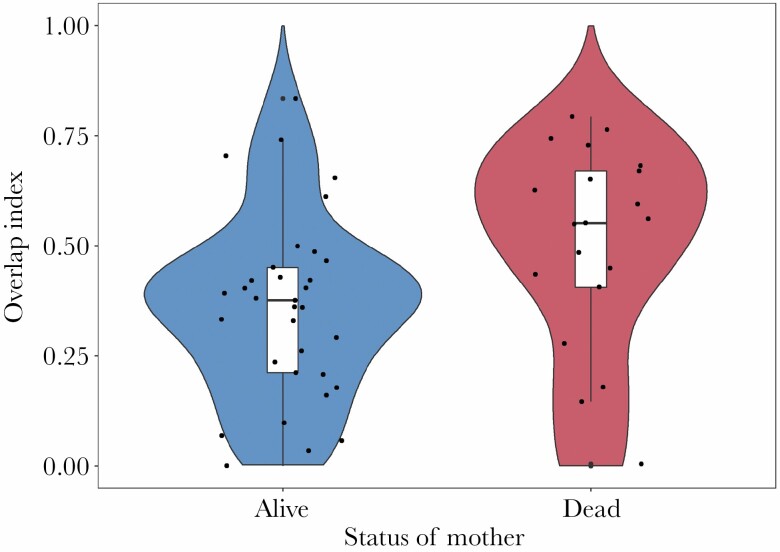
Violin plot showing relationship between the amount of overlap between the settlement home range of a female brown bear and the home range of her mother. The *x* axis indicates whether the mother of the female was alive during the settlement period or had died before settlement. Overlap index values on the *y* axis are between 0 and 1. Mean overlap for females with living mothers was 0.36 (±0.2); mean overlap for females whose mother died before settlement was 0.49 (±0.24).

The overlap index between focal females SHR and their mothers’ home ranges averaged 0.41 ± 0.23 (range 0–0.83). Females whose mothers died before the settlement year (*n* = 20) had a higher degree of overlap with their mother’s last known home range (mean: 0.49 ± 0.24) than females whose mothers were alive (*n* = 32) in the settlement period (mean: 0.36 ± 0.2, *W* = 184, *p* = 0.01, Figure 4).

## DISCUSSION

Our study provides evidence that the social environment influences settlement decisions of a large, non-territorial, solitary-living mammal. We found that female brown bears selected SHRs that overlapped their mother’s home range (support P1). When mothers were removed from the landscape, that is, through hunting, female offspring selected SHRs with greater spatial overlap of their mother’s range, as compared with when mothers were alive (support P2). In contrast, we did not find that females selected SHRs that overlapped related females (no support P3). Instead, females selected SHRs that overlapped familiar females (support P4). Lastly, we found evidence that females used density cues when selecting their SHR, but did not select for lower density areas (no support P5).

We found that the strongest predictor of female SHR selection was overlap with the maternal range. In many mammals, females settle in home ranges that overlap their mother’s home range, that is, are philopatric, (Arnaud et al. 2012; Clutton-Brock and Lukas 2012), likely because resource distribution in that area is most familiar and they receive a certain amount of social tolerance from their mother, as suggested by the resident fitness hypothesis (Wiggett and Boag 1992). Studies from small rodents demonstrate clear fitness benefits from philopatry: female bushy-tailed woodrats (*Neotoma cinerea*) who spatially associated with their mothers after independence had increased over-winter survivorship and reduced reproductive failures compared with those that settled in areas lacking their mother (Moses and Millar 1994). Presence of mothers was related to higher survival in female grey mouse lemurs (*Microcebus murinus*), with daughters of present mothers more likely to survive to the breeding season (Lutermann et al. 2006). Familiarity with resources of an area conveys a considerable advantage to an individual establishing an SHR (Waser and Jones 1983), such as foraging spots (Ashbury et al. 2020), or refugia from predators (Gehr et al. 2020). For example, female orangutans (*Pongo pygmaeus*) show high spatial overlap with their maternal range from independence through sexual maturity and benefit from the mother’s high social tolerance and the familiarity with foraging locations (Ashbury et al. 2020). Among Ursids, females often select an SHR that overlaps with their mothers (Powell 1987; McLellan and Hovey 2001; Støen et al. 2005). However, tolerance is dependent on a mother’s ability to recognize her independent offspring (Waser and Jones 1983). Kin recognition, likely based on olfactory cues (Jojola et al. 2012), has been suggested in our study population (Zedrosser et al. 2007; Swenson and Haroldson 2008). An individual’s mother is the most familiar on the landscape other than full siblings. Thus, it is plausible that female brown bears recognize their independent offspring and tolerate home range overlap with them.

Our strongest evidence for the influence of the social environment was the difference in settlement patterns based on whether or not an individual’s mother was present and alive on the landscape during the settlement period. If a focal female’s mother had died before settlement, the settling female established an SHR with greater overlap of her deceased mother’s last known home range. Conversely, for focal females with living mothers in the settlement period, SHR had less overlap with their mother’s range, which indicates a mother-offspring conflict over space (Frank et al. 2017). Similar patterns have been found in solitary rodents (Lutermann et al. 2006; Arnaud et al. 2012; Sakamoto et al. 2015). For example, experimental removal of Japanese wood mice (*Apodemus speciosus*) mothers inhibited natal dispersal in their female offspring, whereby females whose mothers were present dispersed more frequently and longer distances (Sakamoto et al. 2015). In American black bears, females partially overlapped their mother’s home range although she was alive, but took over her home range after her death (Powell 1987). Our results suggest a tradeoff in which mothers may limit home range sharing to maximize their own fitness. Parent-offspring conflict theory (Trivers 1974) dictates that conflict is expected between mothers and their independent daughters. A settling female should attempt to maximize her fitness by selecting an SHR with the greatest possible overlap with her mother’s range, providing she incurs no related costs. Delayed primiparity of philopatric females has been shown in this population, indicating a potential cost of selecting an SHR overlapping their mother (Støen et al. 2006b). A young female may be compensated for this cost by increased survival and higher future reproduction, possibly due to familiarity with the resources available in their mother’s range. Mothers, however, are expected to limit the amount of overlap to any given daughter to increase her inclusive fitness. A mother that allows her independent daughter greater access to the resources in her HR might decrease her own future survival and reproduction (Trivers 1974). It is possible that mothers are tolerating overlap in marginal areas of their home range although maintaining exclusive use of a “core” area, thereby minimizing their fitness costs, however, we did not investigate these characteristics within home ranges.

We further found that familiarity but not relatedness was selected for in SHR and no interaction between being related and familiar. Thus, familiarity alone appears to be a stronger social cue for female bears in settlement decisions. Early associations as juveniles with overlapping females can be important for discrimination of familiar neighbors. Even a short period of association may allow the recognition of familiar individuals, as shown in Grey seals (*Halichoerus grypus*) (Robinson et al. 2015). This highlights the possibility of young individuals gathering information on familiar conspecifics that they can use later in life. In ursids, information about the social environment is most likely obtained via scent cues and olfactory communication (Clapham et al. 2012; Morehouse et al. 2021), but, given females’ extensive home range overlap, it may also be gained through direct social contact. Future research should directly investigate the use of scent cues and rates of direct contact by female bears.

The benefits of nearby kin have been well documented (Clutton-Brock 2009; Dobson et al. 2012). However, it is becoming increasingly clear that familiarity with other individuals, independent of their relatedness, may give fitness benefits (Ylönen et al. 1990; Shier and Swaisgood 2012). Higher reproductive success in female great tits (*Parus major*) was correlated with a higher number of familiar neighbors and whether their nearest neighbor was familiar (Grabowska-Zhang et al. 2011). Familiarity with conspecifics appears to confer fitness benefits for both survival and reproduction for birds and small mammals (Grabowska-Zhang et al. 2011; Shier and Swaisgood 2012; Siracusa et al. 2021), but it is unknown if this occurs in large mammals. Further research is needed to determine whether settling near closely related or familiar females provides enhanced fitness for dispersing females.

Two potential mechanisms may explain why females select SHR overlapping familiar females but not related females. The first mechanism is the “dear enemy” effect in which an individual will exhibit reduced aggression towards their familiar neighbors compared with strangers (Temeles 1994). This is mostly seen in territorial species (Rosell and Bjørkøyli 2002; Benten et al. 2020; Vázquez et al. 2020). Bears are non-territorial, but previous research has indicated that aggression may be a mechanism for explaining the socio-spatial patterns exhibited in brown bears (Støen et al. 2005). Intraspecific aggression in brown bears has been reported in several populations across their geographic range (Miller 1985; McLellan 1994; Swenson et al. 2001). If aggressive encounters are reduced by selecting SHR overlapping familiar neighbors, this benefits the focal female. Brown bear females exhibit strong site fidelity and stable home ranges are conducive to establishing familiarity with neighboring females. The second mechanism is through familiarity-based kin recognition, that is, discrimination of kin based on association or familiarity (Tang-Martinez 2001). This alternative to phenotypic kin recognition is location-dependent; if an individual is adjacent, they are likely related (Mateo 2004). Tolerance behavior towards neighboring females can be maintained in populations that exhibit philopatry due to the probability that neighbors are closely related (Waser and Jones 1983). If familiars are also relatives, there could be an increase in inclusive fitness. Due to the generally philopatric nature of female brown bears, related individuals are typically clustered spatially (Støen et al. 2005). Despite the suggestion of kin recognition in our population, our results do not suggest its occurrence among female brown bears, but there is support for individual recognition through prior association.

We predicted that females would detect density differences on the landscape and select SHR in areas of lower density, as suggested by previous research (Støen et al. 2006a); however, we did not find support for that in our study. Although females did appear to respond to density in this study population, contrary to our prediction, they selected SHR in areas with higher density relative to their natal ranges. Females possibly settle in areas with higher female density because it could indicate higher quality habitat. Although male bears may disperse into areas lacking conspecifics (Zedrosser et al. 2007), this is not seen with females. Additional research investigating the relationship between population density and habitat quality could help shed light on this settlement pattern.

Strikingly, females do not appear to use non-social environmental cues (i.e., habitat types) when selecting SHR at the second order. Human-mediated homogenization of landscapes through large scale forestry in the study area has created consistent cut blocks and regenerating stands (Josefsson et al. 2010). This suggests that important heterogeneity cues for settlement decisions occurs within the social landscape. Changes in the social makeup of this population are largely driven by hunting (Gosselin et al. 2015; Bischof et al. 2018). As adult females are removed from the population via harvest, surviving females will shift their home ranges to “fill in” vacancies left by the deceased female (Frank et al. 2017). This annual variation in the distribution of the population would make sensitivity towards and use of cues regarding the social environment particularly valuable not only for settlement decisions but also when expanding or shifting the home range configuration over time. Our study highlights that the social environment, beyond conspecific presence or density, is an important consideration when describing settlement decisions and dispersal patterns, and that such information is important for solitary-living species.

## Supplementary Material

arab118_suppl_Supplementary_Methods_S1

arab118_suppl_Supplementary_Methods_S2

arab118_suppl_Supplementary_Table_S1

arab118_suppl_Supplementary_Table_S2

arab118_suppl_Supplementary_Table_S3

## Data Availability

Analyses reported in this article can be reproduced using the data provided by Hansen et al. (2021).
