# Supplementary material for: Social environment shapes female settlement decisions in a solitary carnivore
Source: Behav Ecol. 2021 Oct 18;33(1):137–46. doi: 10.1093/beheco/arab118 (PMC8857934; doi:10.1093/beheco/arab118)
Supplement: arab118_suppl_Supplementary_Methods_S1 [file arab118_suppl_supplementary_methods_s1.pdf]

Supplement S1: deriving and modeling habitat variables in resource selection functions of female brown bear settlement home range selection

Methods

We obtained a 10 m resolution landcover raster, the National Landcover Database (NMD), covering all of Sweden from the Swedish Environmental Protection Agency (Naturvårdsverket 2018). Landcover in the NMD was classified by the Swedish Environmental Protection Agency into 25 thematic classes in three hierarchical levels. Details on the NMD and the classification process can be found at http://www.swedishepa.se/State-of-the-environment/Maps-and-map-services/National-Land-Cover-Database/. We selected six landcover classes of interest for our study: 1) anthropogenic features (built environment), 2) cultivated areas, 3) young forest, 4) mature forest, 5) clearcuts, and 6) bogs.

We overlaid each used and available settlement home range (SHR) polygon (centroids with a 10.5 km buffer) with the landcover raster. We extracted all of the landcover data within the SHR polygon using the 'extract' function in the *velox* package (Hunziker 2018). We used a custom function (which can be accessed at https://mhallwor.github.io/_pages/activities_ExtractingRasterValues) to summarize the amount of each landcover class within the SHR polygon. The result was a dataframe containing the total percent cover of each of the six landcover classes of interest for each used and available SHR.

We added percent cover of each landcover class to our dataframe containing the social variables maternal overlap, relatedness ratio, familiarity index, and density difference that were previously calculated (see main text for details) in each used and available SHR. We import this dataframe below for performing resource selection function modeling and model selection in this supplement.

Load required packages:

```
library(car)
library(glmmTMB)
library(MuMIn)
library(AICcmodavg)
```

Import and prep data:

```
# load data frame containing social and habitat variables

combinedDF <- readRDS("objects/distAdjustHab.rds")

head(combinedDF)
```

```
##   focalID mother used        built         cult matureForest   clearcut
## 1   W0010  W9403    0 0.0011079662 0.0041933951    0.5844815 0.11326936
```

```
## 2   W0010  W9403    0 0.0004824466 0.0008474918    0.5415143 0.08147845
## 3   W0010  W9403    0 0.0009026761 0.0008696514    0.5001504 0.12050909
## 4   W0010  W9403    0 0.0002072877 0.0016876523    0.6635794 0.11157216
## 5   W0010  W9403    0 0.0002017819 0.0013299263    0.6551969 0.11181653
## 6   W0010  W9403    1 0.0004751866 0.0008494649    0.6426707 0.11990849
##   youngForest       bog     famIx  relRatio matOver densDiff
## 1  0.10975652 0.1075296 0.3684211 0.7222222       1        6
## 2  0.18679674 0.1845404 0.2727273 0.3636364       0       -2
## 3  0.15462915 0.2147048 0.2500000 0.4545455       1       -1
## 4  0.04255452 0.1058653 0.2500000 0.6000000       1        3
## 5  0.03469548 0.1159035 0.2857143 0.6923077       1        1
## 6  0.05237144 0.1348704 0.2500000 0.6000000       1        3
```

```
# standardize continuous variables to mean of zero and standard deviation of one

combinedDF[ , c(4:11,13) ]<- scale(combinedDF[ , c(4:11,13)])

head(combinedDF)
```

```
##   focalID mother used      built       cult matureForest    clearcut
## 1   W0010  W9403    0 -0.3734486 -0.2648155   0.30803922  0.09621241
## 2   W0010  W9403    0 -0.4303277 -0.5497270  -0.05917673 -0.77266548
## 3   W0010  W9403    0 -0.3921158 -0.5478400  -0.41268907  0.29408164
## 4   W0010  W9403    0 -0.4553482 -0.4781854   0.98404243  0.04982609
## 5   W0010  W9403    0 -0.4558489 -0.5086466   0.91240235  0.05650509
## 6   W0010  W9403    1 -0.4309879 -0.5495590   0.80534795  0.27766638
##     youngForest         bog       famIx  relRatio matOver    densDiff
## 1  0.0008776213 -0.46361993  0.09736696 1.5950891       1  1.45939654
## 2  1.5315850141  0.77749239 -0.30902781 0.2401396       0 -0.07356864
## 3  0.8924491631  1.26362381 -0.40554657 0.5836479       1  0.11805201
## 4 -1.3343549496 -0.49044124 -0.40554657 1.1332612       1  0.88453460
## 5 -1.4905056413 -0.32866475 -0.25387423 1.4820543       1  0.50129330
## 6 -1.1393031643 -0.02299268 -0.40554657 1.1332612       1  0.88453460
```

Check predictor variables for collinearity:

```
vif(lm(used ~ built + cult + matureForest + clearcut + youngForest + bog
       + matOver + relRatio + famIx + densDiff,
       data = combinedDF))
```

```
##        built         cult matureForest     clearcut  youngForest          bog
##     2.860013     3.094965     1.286195     1.401453     1.573142     1.659896
##      matOver     relRatio        famIx     densDiff
##     1.136876     1.122107     1.197251     1.360491
```

built & cult have VIF > 2. All remaining predictor variables have VIF values < 2.

Check for correlation between built & cult:

```
cor(combinedDF$built, combinedDF$cult)
```

```
## [1] 0.7873743
```

built & cult are too highly correlated to fit in the same model. Variables will be modeled separately.

Fit full model with social and habitat models:

```
combined.rsfA <- glmmTMB(used ~  built +  matureForest + clearcut +
                        youngForest + bog + matOver + relRatio +
                          famIx + densDiff + (1|focalID),
                        family = binomial(),
                        data = combinedDF,
                        na.action = na.fail)
summary(combined.rsfA)
```

```
##  Family: binomial  ( logit )
## Formula:          used ~ built + matureForest + clearcut + youngForest + bog +
##     matOver + relRatio + famIx + densDiff + (1 | focalID)
## Data: combinedDF
##
##      AIC      BIC   logLik deviance df.resid
##      280      322     -129      258      325
##
## Random effects:
##
## Conditional model:
##  Groups  Name        Variance  Std.Dev.
##  focalID (Intercept) 1.257e-09 3.546e-05
## Number of obs: 336, groups:  focalID, 56
##
## Conditional model:
##              Estimate Std. Error z value Pr(>|z|)
## (Intercept)  -2.45212    0.28283  -8.670  < 2e-16 ***
## built        -0.13402    0.24764  -0.541  0.58839
## matureForest  0.06486    0.18345   0.354  0.72368
## clearcut     -0.06881    0.19135  -0.360  0.71914
## youngForest   0.38120    0.19994   1.907  0.05658 .
## bog          -0.31810    0.22050  -1.443  0.14912
## matOver       1.00977    0.33582   3.007  0.00264 **
## relRatio     -0.15599    0.17262  -0.904  0.36619
## famIx         0.83097    0.19275   4.311 1.63e-05 ***
## densDiff      0.73993    0.19756   3.745  0.00018 ***
## ---
## Signif. codes:  0 '***' 0.001 '**' 0.01 '*' 0.05 '.' 0.1 ' ' 1
```

```
# Pseudo R Squared for GLMMs

r.squaredGLMM(combined.rsfA)
```

```
##                   R2m       R2c
## theoretical 0.3127530 0.3127530
## delta       0.1721432 0.1721432
```

```r
combined.rsfB <- glmmTMB(used ~  cult + matureForest + clearcut +
                         youngForest + bog + matOver + relRatio +
                           famIx + densDiff + (1|focalID),
                         family = binomial(),
                         data = combinedDF,
                         na.action = na.fail)
summary(combined.rsfB)
```

```
##  Family: binomial  ( logit )
## Formula:
## used ~ cult + matureForest + clearcut + youngForest + bog + matOver +
##     relRatio + famIx + densDiff + (1 | focalID)
## Data: combinedDF
##
##      AIC      BIC   logLik deviance df.resid
##    278.9    320.9   -128.4    256.9      325
##
## Random effects:
##
## Conditional model:
##  Groups  Name        Variance  Std.Dev.
##  focalID (Intercept) 7.381e-10 2.717e-05
## Number of obs: 336, groups:  focalID, 56
##
## Conditional model:
##              Estimate Std. Error z value Pr(>|z|)
## (Intercept)  -2.48805    0.28771  -8.648  < 2e-16 ***
## cult         -0.33436    0.29609  -1.129 0.258801
## matureForest  0.06560    0.18072   0.363 0.716632
## clearcut     -0.04429    0.18760  -0.236 0.813347
## youngForest   0.35675    0.19978   1.786 0.074147 .
## bog          -0.37323    0.22441  -1.663 0.096270 .
## matOver       1.02510    0.33630   3.048 0.002302 **
## relRatio     -0.17442    0.17296  -1.008 0.313246
## famIx         0.82318    0.19354   4.253 2.11e-05 ***
## densDiff      0.71792    0.19757   3.634 0.000279 ***
## ---
## Signif. codes:  0 '***' 0.001 '**' 0.01 '*' 0.05 '.' 0.1 ' ' 1
```

```r
# Pseudo R Squared for GLMMs

r.squaredGLMM(combined.rsfB)
```

```
##                   R2m       R2c
## theoretical 0.3391719 0.3391719
## delta       0.1899676 0.1899676
```

Fit model with only habitat variables:

```
hab.rsfA <- glmmTMB(used ~  built + matureForest + clearcut +
                        youngForest + bog + (1|focalID),
                        family = binomial(),
                        data = combinedDF,
                        na.action = na.fail)
summary(hab.rsfA)
```

```
##  Family: binomial  ( logit )
## Formula:          used ~ built + matureForest + clearcut + youngForest + bog +
##     (1 | focalID)
## Data: combinedDF
##
##      AIC      BIC   logLik deviance df.resid
##    310.6    337.3   -148.3    296.6      329
##
## Random effects:
##
## Conditional model:
##  Groups  Name        Variance  Std.Dev.
##  focalID (Intercept) 5.262e-10 2.294e-05
## Number of obs: 336, groups:  focalID, 56
##
## Conditional model:
##              Estimate Std. Error z value Pr(>|z|)
## (Intercept)   -1.6598     0.1534 -10.823   <2e-16 ***
## built         -0.2766     0.2120  -1.305   0.1921
## matureForest   0.1105     0.1587   0.696   0.4863
## clearcut      -0.1334     0.1705  -0.782   0.4341
## youngForest    0.2782     0.1801   1.545   0.1223
## bog           -0.4344     0.2018  -2.152   0.0314 *
## ---
## Signif. codes:  0 '***' 0.001 '**' 0.01 '*' 0.05 '.' 0.1 ' ' 1
```

```
# Pseudo R Squared for GLMMs

r.squaredGLMM(hab.rsfA)
```

```
##                    R2m        R2c
## theoretical 0.04677020 0.04677020
## delta       0.02192748 0.02192748
```

```
hab.rsfB <- glmmTMB(used ~  cult + matureForest + clearcut +
                        youngForest + bog + (1|focalID),
                        family = binomial(),
                        data = combinedDF,
                        na.action = na.fail)
summary(hab.rsfB)
```

```
##  Family: binomial  ( logit )
## Formula:
## used ~ cult + matureForest + clearcut + youngForest + bog + (1 |      focalID)
## Data: combinedDF
```

```
##
##      AIC      BIC   logLik deviance df.resid
##    308.4    335.1   -147.2    294.4      329
##
## Random effects:
##
## Conditional model:
##  Groups  Name        Variance  Std.Dev.
##  focalID (Intercept) 6.411e-10 2.532e-05
## Number of obs: 336, groups:  focalID, 56
##
## Conditional model:
##              Estimate Std. Error z value Pr(>|z|)
## (Intercept)  -1.68537    0.15785 -10.677   <2e-16 ***
## cult         -0.43349    0.24405  -1.776   0.0757 .
## matureForest  0.09226    0.15834   0.583   0.5601
## clearcut     -0.08761    0.16765  -0.523   0.6012
## youngForest   0.24305    0.18027   1.348   0.1776
## bog          -0.47299    0.20527  -2.304   0.0212 *
## ---
## Signif. codes:  0 '***' 0.001 '**' 0.01 '*' 0.05 '.' 0.1 ' ' 1
```

```
# Pseudo R Squared for GLMMs

r.squaredGLMM(hab.rsfB)
```

```
##                    R2m        R2c
## theoretical 0.07290817 0.07290817
## delta       0.03468707 0.03468707
```

Fit model with only social variables:

```
soc.rsf <- glmmTMB(used ~  matOver + relRatio + famIx
                   + densDiff + (1|focalID),
                        family = binomial(),
                        data = combinedDF,
                        na.action = na.fail)
summary(soc.rsf)
```

```
##  Family: binomial  ( logit )
## Formula:          used ~ matOver + relRatio + famIx + densDiff + (1 | focalID)
## Data: combinedDF
##
##      AIC      BIC   logLik deviance df.resid
##    274.2    297.1   -131.1    262.2      330
##
## Random effects:
##
## Conditional model:
##  Groups  Name        Variance  Std.Dev.
```

```
##  focalID (Intercept) 7.999e-10 2.828e-05
## Number of obs: 336, groups:  focalID, 56
##
## Conditional model:
##             Estimate Std. Error z value Pr(>|z|)
## (Intercept)  -2.4110     0.2748  -8.774  < 2e-16 ***
## matOver       1.0633     0.3306   3.216 0.001298 **
## relRatio     -0.1804     0.1666  -1.083 0.278703
## famIx         0.8068     0.1850   4.361  1.3e-05 ***
## densDiff      0.6730     0.1807   3.724 0.000196 ***
## ---
## Signif. codes:  0 '***' 0.001 '**' 0.01 '*' 0.05 '.' 0.1 ' ' 1
```

```
# Pseudo R Squared for GLMMs

r.squaredGLMM(soc.rsf)
```

```
##                   R2m       R2c
## theoretical 0.2687401 0.2687401
## delta       0.1437782 0.1437782
```

Model selection

Check for most supported model from the five models.

```
mList <- list(combined.rsfA, combined.rsfB, hab.rsfA, hab.rsfB, soc.rsf)

aictab(mList)
```

```
##
## Model selection based on AICc:
##
##       K   AICc Delta_AICc AICcWt Cum.Wt      LL
## Mod5  6 274.48       0.00   0.90   0.90 -131.11
## Mod2 11 279.71       5.23   0.07   0.96 -128.45
## Mod1 11 280.80       6.32   0.04   1.00 -128.99
## Mod4  7 308.75      34.27   0.00   1.00 -147.20
## Mod3  7 310.93      36.45   0.00   1.00 -148.29
```

“‘

The model containing only social variables has the highest support.

Conclusion

Modeling habitat variables alone in a resource selection function explains very little variation in female brown bear settlement patterns, as evidenced by the pseudo r squared values of 0.05 and 0.08. Combining habitat variables with social variables in a resource selection function did not result in greater model support; the delta AICc was greater than 5 from the model only containing social variables. Due to the lack of explanatory power and support after including habitat variables, we move forward in our analysis using only social variables.

References

Hunziker, P, 2018. velox: fast raster manipulation and extraction. R package version 0.2.0.9002.

Naturvårdsverket, 2018. National Land Cover Database. http://www.swedishepa.se/State-of-the-environment/Maps-and-map-services/National-Land-Cover-Database/
