# Supplementary material for: Social environment shapes female settlement decisions in a solitary carnivore
Source: Behav Ecol. 2021 Oct 18;33(1):137–46. doi: 10.1093/beheco/arab118 (PMC8857934; doi:10.1093/beheco/arab118)
Supplement: arab118_suppl_Supplementary_Methods_S2 [file arab118_suppl_supplementary_methods_s2.pdf]

Supplement S2: checking relationship between familiarity index and relatedness ratio

Due to the potential overlap in the variables 'familiarity index' and 'relatedness ratio' (i.e. a female bear can be both familiar and related), we conducted analyses to determine if these variables were appropriate to include together in the same model.

The first step was to check for collinearity between the two variables. We did this via Spearman's rank correlation. The second step was to create a two-way frequency to look for patterns between occurrences of the variables. Finally, we included an interaction term for these two variables in the model and assessed the results. The procedures and results are shown below.

```
library(tidyverse)
library(rstatix)
library(glmmTMB)
```

Import data and create a subset containing only "used" sites

```
socialRSF <- readRDS("objects/distAdjSocialDF.rds")

usedSHR <- socialRSF %>% filter(used == 1)
```

1. Spearman's rank correlation between familiarity index and relatedness ratio

```
socialRSF %>% cor_test(.,famIx, relRatio, method = "spearman")
```

```
## # A tibble: 1 x 6
##   var1  var2       cor statistic         p method
##   <chr> <chr>    <dbl>     <dbl>     <dbl> <chr>
## 1 famIx relRatio  0.22  4915098. 0.0000385 Spearman
```

2. Create a two-way frequency table to compare co-occuring values for familiarity index and relatedness ratio

```r
binnedDF <- usedSHR %>%
  mutate(famBin = case_when(famIx == 0 ~ "none",
                            famIx > 0 & famIx < 0.25 ~ "low",
                            famIx >= 0.25 & famIx < 0.65 ~ "medium",
                            TRUE ~ "high"),
         relBin = case_when(relRatio == 0 ~ "none",
                            relRatio > 0 & relRatio < 0.25 ~ "low",
                            relRatio >= 0.25 & relRatio < 0.65 ~ "medium",
                            TRUE ~ "high"))



table(binnedDF$famBin, binnedDF$relBin, dnn = c("famIx", "relRatio"))
```

```
##         relRatio
## famIx    high low medium none
##   high      1   1      6    3
##   low       2   0      2    2
##   medium    2   9     20    6
##   none      0   0      1    1
```

3. Add interaction term between relatedness ratio and familiarity index

```r
int.glmm <- glmmTMB(used ~  momPresent + z.densDiff +
                            z.famIx * z.relRatio + (1|focalID),
                            family = binomial(),
                            data = socialRSF,
                            na.action = na.fail)
summary(int.glmm)
```

```
##  Family: binomial  ( logit )
## Formula:          used ~ momPresent + z.densDiff + z.famIx * z.relRatio + (1 |
##     focalID)
## Data: socialRSF
##
##      AIC      BIC   logLik deviance df.resid
##    276.1    302.8   -131.0    262.1      329
##
## Random effects:
##
## Conditional model:
##  Groups  Name        Variance  Std.Dev.
##  focalID (Intercept) 8.745e-10 2.957e-05
## Number of obs: 336, groups:  focalID, 56
##
## Conditional model:
##                      Estimate Std. Error z value Pr(>|z|)
## (Intercept)           -2.4266     0.2770  -8.760  < 2e-16 ***
```

```
## momPresent           1.0566     0.3303   3.199 0.001381 **
## z.densDiff           0.7043     0.1940   3.631 0.000283 ***
## z.famIx              0.8343     0.1977   4.220 2.45e-05 ***
## z.relRatio          -0.2129     0.1848  -1.152 0.249320
## z.famIx:z.relRatio   0.0694     0.1726   0.402 0.687597
## ---
## Signif. codes:  0 '***' 0.001 '**' 0.01 '*' 0.05 '.' 0.1 ' ' 1
```

Conclusion

The three analyses we performed did not indicate a relationship between relatedness ratio and familiarity index. The Spearman's rho statistic was only 0.22, there was no clear pattern in the two-way frequency table, and including an interaction term in the model for the two variables indicated no relationship between relatedness ratio and familiarity index. We thus feel confident that any overlap of individuals in the relatedness or familiarity variables was not influential in this study.
