# Supplementary material for: Social environment shapes female settlement decisions in a solitary carnivore
Source: Behav Ecol. 2021 Oct 18;33(1):137–46. doi: 10.1093/beheco/arab118 (PMC8857934; doi:10.1093/beheco/arab118)
Supplement: arab118_suppl_Supplementary_Table_S1 [file arab118_suppl_supplementary_table_s1.docx]

Table S1. Description of social variables used to evaluate female brown bear selection of settlement home ranges in Sweden, 1998-2018. Statistics are shown for the realized settlement home ranges (SHR; i.e. ‘used’ home ranges from resource selection function). Min = minimum, max = maximum, SD = standard deviation. Maternal overlap is given as the proportion of females that had mothers present on the landscape and had SHR overlapping their mother’s home range (n = 32 present mothers, n = 4 present without overlap, n = 20 absent mothers).

| **Variable** | **Type** | **Min** | **Max** | **Mean** | **SD** |
| --- | --- | --- | --- | --- | --- |
| *Maternal overlap*:  Home range of mother overlaps focal female’s SHR | Binary | 0 | 1 | 0.89 | - |
| *Familiarity Index*:  Proportion of known to total females home ranges overlapping focal females SHR | Continuous | 0 | 1 | 0.44 | 0.22 |
| *Relatedness Ratio*:  Proportion of related to total female home ranges overlapping focal females SHR | Continuous | 0 | 0.83 | 0.3 | 0.23 |
| *Density Difference:*  Difference in density between focal females SHR and natal home range (NHR) SHR density – NHR density | Continuous | -9 | 12 | 0.3 | 4.41 |
