# Supplementary material for: Social environment shapes female settlement decisions in a solitary carnivore
Source: Behav Ecol. 2021 Oct 18;33(1):137–46. doi: 10.1093/beheco/arab118 (PMC8857934; doi:10.1093/beheco/arab118)
Supplement: arab118_suppl_Supplementary_Table_S2 [file arab118_suppl_supplementary_table_s2.docx]

Table S2. Results from resource selection function models of female Scandinavian brown bear settlement home range selection. Interactions were fit individually due to model convergence issues related to sample size. Interactions are shown in italics and significant p-values (≤ 0.05) are shown in bold. None of the interaction terms in the models were significant and the base model had the lowest AICc value, i.e. was the most supported model. Familiarity index, maternal overlap, and density difference were significant in all models.

| **Model** | **Term** | **β** | **Std Error** | **P Value** | **CI** | **AICc** |
| --- | --- | --- | --- | --- | --- | --- |
| Base Model | famIx | 0.806 | 0.185 | **< 0.001** | 0.44 – 1.17 | **274.5** |
|  | relRatio | -0.185 | 0.171 | 0.279 | -0.52 – 0.15 |  |
|  | matOver | 1.063 | 0.331 | **0.001** | 0.42 – 1.71 |  |
|  | densDiff | 0.678 | 0.182 | **< 0.001** | 0.32 – 1.04 |  |
| Interaction 1 | famIx | 0.801 | 0.185 | **< 0.001** | 0.44 – 1.16 | 276.5 |
|  | relRatio | -0.251 | 0.278 | 0.366 | -0.8 – 0.29 |  |
|  | matOver | 1.068 | 0.333 | **0.001** | 0.42 – 1.72 |  |
|  | densDiff | 0.677 | 0.182 | **<** **0.001** | 0.32 – 1.03 |  |
|  | *matOver:relRatio* | 0.109 | 0.355 | 0.758 | -0.59 – 0.8 |  |
| Interaction 2 | famIx | 0.785 | 0.221 | **< 0.001** | 0.35 – 1.22 | 276.5 |
|  | relRatio | -0.188 | 0.172 | 0.275 | -0.53 – 0.15 |  |
|  | matOver | 1.045 | 0.347 | **0.003** | 0.36 – 1.72 |  |
|  | densDiff | 0.685 | 0.186 | **< 0.001** | 0.32 – 1.05 |  |
|  | *matOver:famIx* | 0.057 | 0.336 | 0.865 | -0.6 – 0.72 |  |
| Interaction 3 | famIx | 0.811 | 0.187 | **< 0.001** | 0.44 – 1.18 | 275.8 |
|  | relRatio | -0.19 | 0.172 | 0.269 | -0.53 – 0.15 |  |
|  | matOver | 1.139 | 0.343 | **< 0.001** | 0.47 – 1.81 |  |
|  | densDiff | 0.851 | 0.274 | **0.002** | 0.31 – 1.39 |  |
|  | *matOver:densDiff* | -0.279 | 0.326 | 0.391 | -0.92 – 0.36 |  |
| Interaction 4 | famIx | 0.834 | 0.198 | **< 0.001** | 0.45 – 1.22 | 276.4 |
|  | relRatio | -0.213 | 0.185 | 0.249 | -0.57 – 0.15 |  |
|  | matOver | 1.057 | 0.33 | **0.001** | 0.41 – 1.7 |  |
|  | densDiff | 0.704 | 0.194 | **< 0.001** | 0.32 – 1.08 |  |
|  | *famIx:relRatio* | 0.069 | 0.173 | 0.688 | -0.27 – 0.41 |  |
| Interaction 5 | famIx | 0.827 | 0.186 | **< 0.001** | 0.46 – 1.19 | 275.1 |
|  | relRatio | -0.157 | 0.171 | 0.36 | -0.49 – 0.18 |  |
|  | matOver | 1.064 | 0.332 | **0.001** | 0.41 – 1.71 |  |
|  | densDiff | 0.681 | 0.186 | **< 0.001** | 0.32 – 1.05 |  |
|  | *famIx:densDiff* | 0.205 | 0.174 | 0.238 | -0.14 – 0.55 |  |
| Interaction 6 | famIx | 0.809 | 0.186 | **< 0.001** | 0.44 – 1.17 | 276.4 |
|  | relRatio | -0.166 | 0.175 | 0.341 | -0.51 – 0.18 |  |
|  | matOver | 1.053 | 0.331 | **0.001** | 0.41 – 1.7 |  |
|  | densDiff | 0.714 | 0.201 | **< 0.001** | 0.32 – 1.11 |  |
|  | *relRatio:densDiff* | -0.086 | 0.195 | 0.659 | -0.47 – 0.3 |  |
