# Supplementary material for: Social environment shapes female settlement decisions in a solitary carnivore
Source: Behav Ecol. 2021 Oct 18;33(1):137–46. doi: 10.1093/beheco/arab118 (PMC8857934; doi:10.1093/beheco/arab118)
Supplement: arab118_suppl_Supplementary_Table_S3 [file arab118_suppl_supplementary_table_s3.docx]

Table S3. Results of adding an additional random intercept for year since family breakup in a resource selection function for female Scandinavian brown bear settlement home range selection. The original base model is shown first, with the model containing the random intercept below (Year Model). Variable coefficients (β), standard error (SE), 95% confidence intervals (CI), p-values, and AICc values for each model are presented. Significant p-values are in bold. Variance for the random intercept for year was < 0.001. Standard deviation for the random intercept for year was < 0.001.

| **Model** | **Term** | **β** | **SE** | **P Value** | **CI** | **AICc** |
| --- | --- | --- | --- | --- | --- | --- |
| Base Model | famIx | 0.806 | 0.185 | **< 0.001** | 0.44 – 1.17 | 274.5 |
|  | relRatio | -0.185 | 0.171 | 0.279 | -0.52 – 0.15 |  |
|  | matOver | 1.063 | 0.331 | **0.001** | 0.42 – 1.71 |  |
|  | densDiff | 0.678 | 0.182 | **< 0.001** | 0.32 – 1.04 |  |
| Year Model | famIx | 0.806 | 0.185 | **< 0.001** | 0.44 – 1.17 | 276.6 |
|  | relRatio | -0.185 | 0.171 | 0.279 | -0.52 – 0.15 |  |
|  | matOver | 1.063 | 0.331 | **0.001** | 0.42 – 1.71 |  |
|  | densDiff | 0.678 | 0.182 | **< 0.001** | 0.32 – 1.04 |  |
